# Increased Mortality Among Young Systemic Sclerosis Patients During the COVID-19 Pandemic: A Nationwide Data Analysis from Thailand

**DOI:** 10.3390/life16020201

**Published:** 2026-01-26

**Authors:** Chingching Foocharoen, Patnarin Pongkulkiat, Tippawan Onchan, Siraphop Suwannaroj, Sarrote Boonkerd, Plumekamol Tangwattanakunchai, Ajanee Mahakkanukrauh

**Affiliations:** 1 Department of Medicine, Faculty of Medicine, Khon Kaen University, Khon Kaen 40002, Thailand; fching@kku.ac.th (C.F.);; 2 Information and Communication Technology Center Office of the Permanent Secretary for Public Health, Meuang, Nonthaburi 11000, Thailand

**Keywords:** rheumatology, epidemiology, health, public health, COVID-19

## Abstract

**Background:** Beyond the direct COVID-19 effects, the pandemic’s broader impact on vulnerable groups, such as patients with systemic sclerosis (SSc), is particularly concerning, especially regarding any resulting increase in overall mortality due to healthcare access disruptions. We aimed to determine excess all-cause mortality in SSc patients before and during the pandemic. **Methods:** We examined mortality data from Thailand’s Ministry of Public Health database for adults with SSc (ICD-10: M34). According to the WHO methodology, a negative binomial distribution model was used to estimate the expected number of deaths using pre-pandemic data (1 January 2015–31 December 2019). We evaluated actual versus expected deaths during the pandemic (1 January 2020 to 31 December 2022), defining excess mortality as the difference between observed and projected deaths under normal conditions. **Results:** The total number of all-cause deaths in Thailand was 2,325,384 in the pre-pandemic period and 1,634,121 during the pandemic period. The mortality rate among patients with SSc was 3693 before and 3107 during the pandemic. Of those with SSc, 1785 of the deceased were female, and the observed mortality was significantly lower than expected, with an excess death count of −368 (95% CI: −459 to −277), as well as in males with an excess death count of −123 (95% CI: −198 to −48). However, younger SSc patients (aged 18–29 years) experienced significantly higher excess mortality, with an excess death count of 11 (95% CI: 4–18). **Conclusions:** During the COVID-19 pandemic, neither sex had significantly higher SSc mortality; however, mortality in younger SSc patients increased significantly compared to pre-pandemic levels, underscoring the need for tailored therapies.

## 1. Introduction

Systemic sclerosis (SSc) is a rare, chronic autoimmune connective tissue disease characterized by vasculopathy, immune dysregulation, and progressive fibrosis of the skin and internal organs [1]. SSc carries a high risk of serious complications, including interstitial lung disease, pulmonary arterial hypertension, scleroderma renal crisis, and severe gastrointestinal problems [1]. Ongoing clinical evaluations and proactive management are crucial for enhancing outcomes and improving quality of life.

The coronavirus 2 (SARS-CoV-2) has posed significant challenges to patients with autoimmune rheumatic diseases, including SSc. During the pandemic, the enormous strain on healthcare resources likely compromised the delivery of essential care, putting patients with SSc at greater risk for disease progression and poor outcomes [2]. Severe acute respiratory syndrome SARS-CoV-2, the virus that causes coronavirus disease 2019 (COVID-19), emerged in late 2019 and rapidly spread worldwide, resulting in a global pandemic that significantly impacted health systems [3]. Between 2019 and 2021, the effects of the pandemic were profoundly felt in Thailand, creating unprecedented challenges in healthcare delivery and limiting access to routine medical services [4]. These challenges were especially difficult for people living with chronic illnesses, including SSc. Effective management of SSc depends on consistent long-term medical follow-up, early detection of complications, and timely treatment interventions to slow disease progression.

As COVID-19 cases increased, healthcare systems worldwide, including those in Thailand, struggled to meet the demand for patient care [4]. Hospitals redirected staff and resources to pandemic response efforts, leading to widespread disruptions in outpatient care, postponed non-urgent appointments, and fewer opportunities for routine health monitoring. For patients with chronic illnesses such as SSc, these disruptions meant delayed diagnoses, interrupted treatments, and missed chances to catch early signs of worsening disease [5]. Emmi et al. [6] reported that no elevated risk of SARS-CoV-2 infection or associated complications was observed in patients with SSc compared to the general population. Gianfrancesco et al. [7] also found that the risk factors for hospitalization and severe COVID-19 outcomes were related to older age, high-dose glucocorticoid use, and the presence of comorbidities rather than the diagnosis of SSc itself. However, these studies included SSc as a minority autoimmune and rheumatic disease population. However, Chaisrimaneepan et al. [8] and Bournia et al. [9], analyzing nationwide databases from the USA and Greece, respectively, found that patients with SSc and COVID-19 had a higher risk of hospitalization complications and mortality than those without non-SSc patients. These contrasting results highlight the uncertainty regarding COVID-19 outcomes in patients with SSc.

Between January 2020 and December 2023, Thailand reported over 4.7 million confirmed COVID-19 cases and 34,506 related deaths [10]. However, beyond the direct impact of COVID-19, little is known about how the pandemic affected vulnerable groups, such as those living with SSc. Specifically, it remains unclear whether disruptions in healthcare access led to an increase in overall mortality among these patients. We hypothesized that disruptions in healthcare and the direct impact of COVID-19 have led to increased mortality among patients with SSc. To address this knowledge gap, we examined whether there was an excess in all-cause mortality among individuals diagnosed with SSc during the COVID-19 pandemic in Thailand compared to before the pandemic. We focused our analysis on national mortality data from 1 January 2020, through 31 December 2022—a timeframe that captures the span from the pandemic’s emergence to its peak, up until the Thai government lifted its COVID-19 Emergency Decree and reclassified the disease from a “dangerous communicable disease” to one “under surveillance” [11].

## 2. Methods

### 2.1. Study Design

This retrospective study analyzed data from two distinct periods: the pre-COVID-19 period (1 January 2015 to 31 December 2019) and the COVID-19 period (1 January 2020 to 31 December 2022). Data were obtained from the Information and Communication Technology Center, Ministry of Public Health, Thailand, which integrates healthcare records from multiple sources: (i) the National Health Security Office (NHSO), (ii) the Civil Servants Benefit System under the Comptroller General’s Department, (iii) the Social Security Office, and (iv) self-paid healthcare services.

The study included all patients aged 18 years or older with a primary diagnosis of systemic sclerosis (SSc), classified under ICD-10 code M34. The dataset comprised variables such as patient age, sex, year of healthcare visit, hospital name, district (amphur), province, primary diagnosis, comorbidities, survival status (alive or deceased), cause of death, and the date of death. The data were stratified by province and region for analysis. In addition to SSc-specific data, all-cause mortality records during the study period were categorized by province and region. Deaths attributed to COVID-19 during the pandemic were also captured and analyzed separately.

### 2.2. Definitions

Excess mortality was defined as the difference between the observed number of deaths during a crisis period and the number of deaths expected under normal, non-crisis conditions. All-cause mortality referred to the total number of deaths recorded from all causes without attribution to a specific disease or condition. Expected deaths represented the estimated number of deaths that would have occurred in the absence of the COVID-19 pandemic, typically derived from historical mortality trends. The P-score was the standardized measure of excess mortality calculated as the ratio of excess deaths to expected deaths, expressed as a percentage. It quantified the relative increase in mortality and facilitated comparisons between periods and geographic regions.

### 2.3. Statistical Analysis

All-cause mortality data from 1 January 2015 to 31 December 2019 were used to estimate the counterfactual baseline mortality in the absence of the COVID-19 pandemic. Mortality counts were modeled within a generalized additive regression framework using either a Poisson or negative binomial distribution, with the latter applied when overdispersion was present. Let $Y_{c,t}$ denote the observed mortality count in a country/region c at time t. The expected mortality $\left(E_{c,t}\right)$ was estimated as [12]:

$$Y_{c,t}∼\mathrm{Negative}\;\mathrm{Binomial}\left(\lambda_{c,t}\right)$$

$$\log\left(\lambda_{c,t}\right)=\alpha +f_{1}\left(\mathrm{time}\right)+f_{2}\left(\mathrm{season}\right)+\beta_{1}\left(\mathrm{age}\right)+\beta_{2}\left(\mathrm{sex}\right)+\beta_{3}\left(\mathrm{region}\right)$$

Age group, sex, and geographic region were included as covariates. Long-term secular trends were modeled using non-cyclic cubic regression splines, and seasonal variation was captured using penalized cyclic cubic splines with a 52-week periodicity. Smoothing parameters were estimated via restricted maximum likelihood and optimized using generalized cross-validation. This approach aligns with the WHO Western Pacific Region excess mortality framework.

Excess mortality was calculated as the difference between observed and expected mortality counts $\left(Y_{c,t}-E_{c,t}\right)$. To facilitate comparability across regions, P-scores were computed to express excess mortality as the percentage deviation from baseline expectations:

$$P{\text{-}\mathrm{score}}_{c,t}=\frac{Y_{c,t}-E_{c,t}}{E_{c,t}}\times 100$$

Model validity was assessed through out-of-sample performance testing by fitting models to pre-pandemic data (2015–2018) and forecasting mortality for 2019. The predictive accuracy, 95% prediction interval coverage, dispersion parameters, and residual diagnostics were examined. This modeling strategy has previously demonstrated robust coverage performance across the age and sex strata. Analyses were conducted at the national and regional levels and stratified by province and month to explore geographical and temporal heterogeneity. The spatial distribution of all causes of death in Thailand were analysed and presented using QGIS software version 3.36.2: an open source and free software of geographic data system information was used for generating the maps; https://www.qgis.org/en/site/ (accessed on 26 May 2025). All data analyses were performed using R version 4.4.0 (R Foundation for Statistical Computing) and STATA version 16.0 (StataCorp., College Station, TX, USA).

## 3. Results

### 3.1. Increased Vulnerability of Elderly and Female SSc Patients During COVID-19, with Heart Failure and Pneumonia as Leading Causes of Death

During the pre-COVID-19 pandemic period (1 January 2015 to 31 December 2019), 2,325,384 deaths were recorded in Thailand. In comparison, 1,634,121 deaths were reported during the COVID-19 pandemic (1 January 2020 to 31 December 2022) (Table 1). Throughout the pandemic, mortality patterns shifted notably, with males showing a higher mortality rate (56.62%) than females (43.37%). Age-specific analyses revealed that individuals aged 70 years or older accounted for a higher percentage of deaths during the pandemic than before (52.14% vs. 50.01%), highlighting the increased susceptibility of older adults to COVID-19 and the impact of related disruptions in healthcare services.

Among individuals diagnosed with SSc, 3693 deaths occurred during the pre-pandemic period, and 3107 deaths were recorded during the pandemic. Notably, all-cause mortality among patients with SSc peaked in the later stage of the pandemic (1 January 2022–31 December 2022), whereas a slight reduction in deaths was observed during the early (1 January 2020–31 December 2020) and middle (1 January 2021–31 December 2021) phases.

Female patients with SSc had a higher proportion of all-cause mortality (57.42%) than males (42.54%), which corresponds with the well-documented greater prevalence and disease burden of SSc observed in women. Age distribution analyses indicated that SSc-related mortality was highest among individuals aged 60–69 years (34.04%) during the pandemic, a modest increase from that in the pre-pandemic period (32.81%).

Forty-two deaths among patients with SSc were directly attributed to COVID-19, with approximately two-thirds occurring in females and primarily affecting individuals aged ≥ 70 years. In addition to COVID-19-related mortality, heart failure, and pneumonia remained the leading non-SSc causes of death among patients with SSc throughout the study period.

A comparison of all-cause mortality, SSc-related death, death due to COVID-19, and the five most common causes of non-SSc-related death is presented in Table 2.

### 3.2. SSc Mortality Peaks in Younger Adults During COVID-19

To explore how the COVID-19 pandemic may have influenced age-specific mortality patterns among patients with SSc, we analyzed excess deaths by age group. The analysis revealed that the highest number of SSc-related deaths occurred in the 18–29-year age group compared to the pre-pandemic period, an unexpected finding, given the traditionally higher disease burden among older populations. This pattern suggests a possible shift in the disease burden toward younger individuals during the pandemic (Table 3).

### 3.3. Sex Disparities in SSc-Related Deaths Amid COVID-19

To better comprehend the sex-specific impact of the COVID-19 pandemic on SSc mortality, we analyzed the excess deaths by sex. The analysis revealed that women had a higher mortality rate than men throughout the pandemic. SSc-related deaths were significantly more pronounced among females, as reflected by the p-scores (−17.1 for females vs. −8.5 for males). The highest number of SSc-related deaths in both sexes was recorded between 1 January 2021 and 31 December 2021, indicating that this period marked the peak impact of the pandemic on patients with SSc (Table 4).

### 3.4. Temporal Trends in All-Cause and SSc-Related Mortality from 2015 to 2022

**Figure 1:**
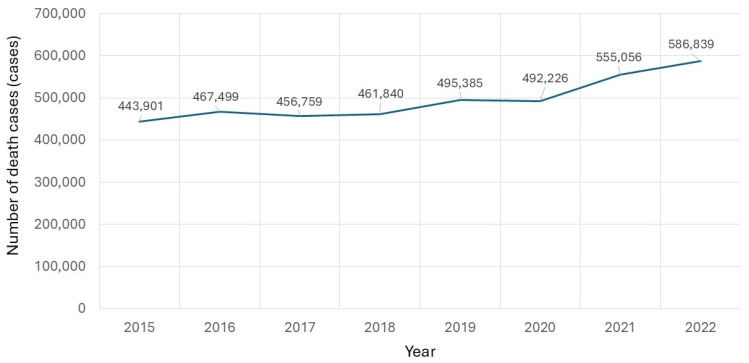
Yearly number of deaths from 2015 to 2022.

**Figure 2:**
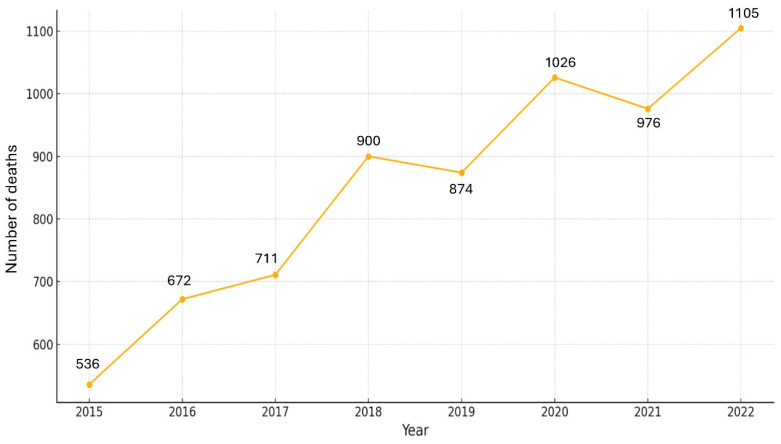
Yearly number of deaths due to SSc from 2015 to 2022.

**Figure 3:**
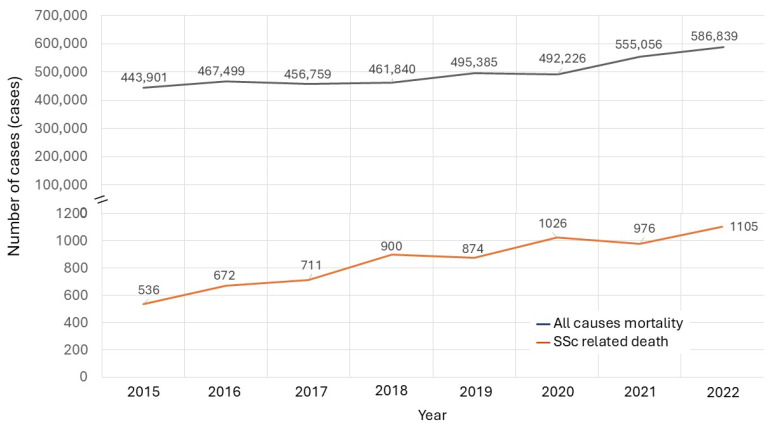
Comparison of yearly deaths due to all causes and SSc from 2015 to 2022.

To determine the impact of the COVID-19 pandemic on overall and disease-specific mortality, particularly among vulnerable populations, we analyzed the trends in all-cause and SSc-related deaths over time. The total all-cause mortality in Thailand from 2015 to 2022 demonstrated a marked increase beginning in 2020, coinciding with the onset of the COVID-19 pandemic (Figure 1). In contrast, SSc-related deaths declined in 2021 compared to 2020 but increased again in 2022 (Figure 2). A comparative analysis of total mortality and SSc-specific deaths further illustrates these trends, showing the distinct impact of the pandemic on patients with SSc (Figure 3). Monthly data on SSc-related deaths from 2015 to 2022 revealed a notable peak at the end of 2020, possibly reflecting early disruptions in care for this vulnerable population during the pandemic.

### 3.5. Seasonal and Temporal Trends in SSc Mortality (2015–2022)

**Figure 4:**
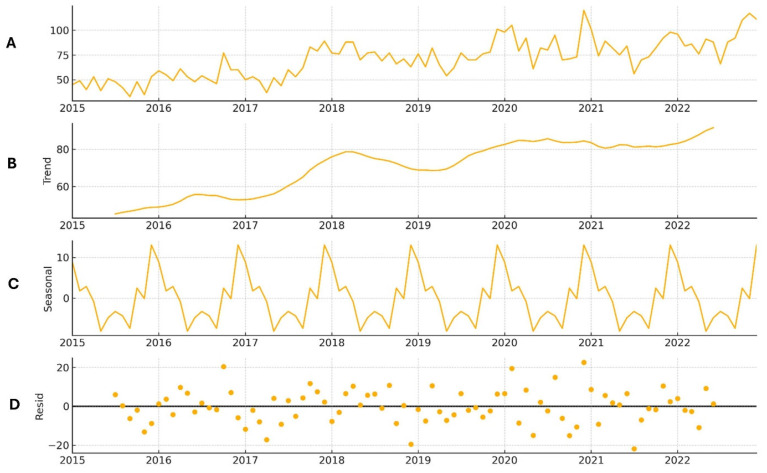
Seasonal decomposition plot of deaths due to SSc from 2015 to 2022. Number of death cases (**A**), trend of death cases (**B**), number of deaths by season (**C**), and residual number of death cases that could not be explained by season (**D**).

To better understand the temporal patterns and underlying factors influencing mortality trends during the study period, we performed seasonal decomposition analyses of deaths from all causes and from systemic sclerosis (SSc) between 2015 and 2022. The seasonal decomposition plots are presented in Figure 4. The graph in Figure 4A illustrates the annual number of deaths, reflecting a combination of long-term trends and seasonal fluctuations. Figure 4B illustrates the long-term trend, highlighting periods of increase and decrease in mortality, independent of seasonal variations. Figure 4C presents the seasonal component, demonstrating recurring annual patterns of mortality, such as higher death rates during the winter or rainy seasons. Finally, Figure 4D displays the residual component, which captures irregular fluctuations that cannot be explained by either trends or seasonality, potentially reflecting specific events such as disease outbreaks or unusual circumstances in specific months.

Regional variations in SSc and all-cause mortality across Thailand during the pandemic.

**Figure 5:**
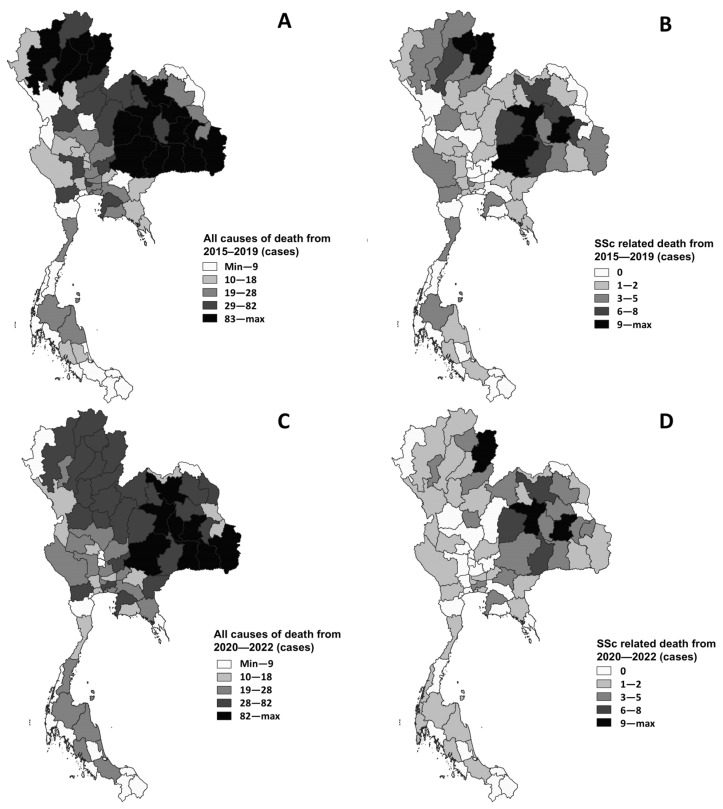
Number of deaths categorized by time and cause. All causes of death from 2015 to 2019 (**A**), SSc-related deaths from 2015—2019 (**B**), all causes of death during the emerging COVID-19 pandemic (**C**), and SSc-related deaths during the emerging COVID-19 pandemic (**D**). (QGIS software version 3.36.2: an open source and free software of geographic data system information was used for generating the maps; https://www.qgis.org/en/site/, accessed on 26 May 2025).

The spatial distribution of all causes of death in Thailand is shown in Figure 5. During the pre-COVID-19 period (2015–2019), the highest number of all-cause deaths was observed in the northeastern and northern regions. This pattern closely aligns with the geographic distribution of SSc-related deaths during the same period, suggesting a potential regional clustering of the SSc burden that may be influenced by demographic, environmental, or healthcare access factors.

Similarly, during the COVID-19 pandemic, the all-cause mortality rates remained elevated in the northern and northeastern provinces. SSc-related deaths during the pandemic also mirrored this spatial trend, indicating that the same regions continued to bear a disproportionate mortality burden. This consistent geographic overlap suggests that populations in these areas may be more vulnerable due to limited healthcare infrastructure, delayed diagnosis, or a higher prevalence of risk factors associated with both SSc and COVID-19 complications. These findings underscore the importance of region-specific public health interventions and resource allocation in improving outcomes for patients with SSc, particularly in the northern and northeastern regions of Thailand. Further investigation into region-specific determinants, including socioeconomic status, healthcare accessibility, and environmental exposure, may help explain the observed spatial disparities.

## 4. Discussion

This nationwide retrospective study examined the impact of the COVID-19 pandemic on all-cause and SSc-related mortality in Thailand between 2015 and 2022. We aimed to determine whether individuals with SSc experienced excess mortality during the pandemic and how these trends varied by age, sex, region, and time. By comparing mortality patterns before and during the pandemic, we aimed to identify the broader, indirect consequences of healthcare disruptions in this vulnerable patient group.

Although SSc-related deaths remained relatively low in absolute numbers, our findings revealed notable shifts in the mortality patterns. Only 42 deaths among patients with SSc were directly attributed to COVID-19, suggesting that the viral burden alone may not have been the primary driver of mortality in this population. This may reflect the effectiveness of public health measures, shielding behaviors, or potentially underreporting [13,14,15]. Nevertheless, heart failure and pneumonia remained the leading causes of death. Both are well-documented complications of SSc [16,17] that may have been exacerbated by disruptions to routine care and delayed medical attention during the pandemic.

However, it is important to acknowledge that while the official mortality registry categorizes heart failure and non-COVID pneumonia as distinct causes, the potential underlying influence of SARS-CoV-2 infection on these outcomes cannot be entirely dismissed. Recent literature has highlighted the complex interplay between chronic cardiomyopathies and persistent viral infections, which may complicate both the diagnosis and clinical management of patients with pre-existing cardiovascular vulnerabilities.

One of the strengths of this study is its use of nationwide population-based data, which allowed us to capture long-term mortality trends across multiple subgroups. To our knowledge, this is the first study to assess excess mortality in patients with SSc during the COVID-19 pandemic using such a large and comprehensive dataset. By spanning 2015 to 2022, we explored both pre- and post-pandemic patterns and seasonal trends, providing an important context for our analysis. This broad perspective allowed us to identify demographic and geographic variations in SSc-related and non-SSc-related deaths.

Among the key findings was the heightened vulnerability of specific patient subgroups. Female patients consistently accounted for the majority of SSc-related deaths, in line with the known female predominance of the disease [18]. However, the P-score analysis showed greater excess mortality among women during the pandemic, indicating a disproportionate impact. This may reflect global trends, where women often face increased barriers to healthcare access due to caregiving responsibilities, socioeconomic stressors, and systemic inequities [19].

We observed increased mortality among younger patients aged 18–29 years during the pandemic. This finding is contrary to our expectations, as this age group typically has a better prognosis than older age groups [20]. This might be related to hidden vulnerabilities, such as delayed diagnosis, inadequate disease monitoring, or limited access to specialized care for early-stage disease [21,22]. The prevalence of SSc mostly occurs in late middle age, with a peak between 60 and 69 years [23]. Several potential explanations may account for this disproportionate mortality burden among younger SSc patients. First, SSc is often perceived as a disease of older adults, potentially leading to diagnostic delays when it presents in younger patients. At this age, they may lack disease awareness, experience delays in seeking specialist care, and subsequent delays in treatment, which may or may not be related to disruptions in healthcare access during the COVID-19 pandemic, ultimately leading to higher mortality. Second, pandemic stressors—job loss, reduced insurance, and healthcare avoidance—amplified these barriers, compromising immediate financial well-being and long-term mental health [24]. Third, young adults may have been less likely to prioritize chronic disease management when facing immediate pandemic-related stressors. While elderly patients and those with obvious high-risk conditions received prioritized attention, young adults with less apparent vulnerability may have experienced greater reductions in routine monitoring, medication adjustments, and health information receiving [25]. These findings suggest that healthcare for SSc should focus more on younger age groups in the event of emerging diseases.

In contrast, SSc in the older age group had significantly lower mortality rates than before the emergence of COVID-19. This reduction may be attributed to the extent and effectiveness of self-protective behaviors adopted by individuals with SSc [9], a group in which a significant proportion has clinically significant interstitial lung disease. This finding demonstrates that adherence to public health measures by vulnerable populations with chronic illnesses can reduce all-cause mortality during a pandemic.

Regional disparities also emerged. Patients in the northern and northeastern regions consistently experienced higher mortality rates, both from all causes and SSc-specific causes. These regions face structural challenges, including lower availability of rheumatology specialists, fewer healthcare facilities, and higher poverty rates [23]. Such disparities were likely magnified during the pandemic, reinforcing the need for region-specific health interventions.

In comparison to this study on SSc, excess mortality during the COVID-19 pandemic has been observed in other connective tissue and chronic diseases, although the extent of the impact varies. Studies have shown that patients with diseases such as systemic lupus erythematosus (SLE), rheumatoid arthritis (RA), and idiopathic inflammatory myopathies experienced increased mortality during the pandemic, primarily due to delays in care, complications from infections, and exacerbations of underlying disease [26,27]. The all-cause mortality rate in patients with RA was reported to be higher than that in the general population, but there was no difference in patients with SLE, ankylosing spondylitis, and psoriatic arthritis [9]. Unlike SSc, where overall mortality did not significantly increase, a study found a more than 50% decrease in all-cause mortality among SSc patients [9]. These conditions often saw more pronounced mortality rates, particularly in patients with comorbidities such as cardiovascular disease [28]. The contrasting trends between SSc and other chronic diseases highlight the heterogeneous nature of pandemic-related outcomes and suggest that different diseases may have different levels of vulnerability to COVID-19, requiring tailored approaches in both care and research.

Despite these valuable insights, this study has several limitations. First, our reliance on ICD-10-coded administrative data means that diagnoses depend on what the attending physician recorded. Although SSc has distinct clinical features, especially its characteristic skin thickening, the potential for coding inconsistencies cannot be completely ruled out. However, the likelihood of widespread misclassification is low. Second, although heart failure was the most frequently reported non-SSc-related cause of death, it is often a direct complication of SSc, particularly due to myocardial fibrosis or pulmonary hypertension. The limitations of ICD-10 coding prevented us from distinguishing whether heart failure in these cases was secondary to SSc or an unrelated condition, potentially leading to an underestimation of the mortality burden associated with SSc in this study. Third, owing to data limitations, we lacked data on specific antibodies, SSc subsets, skin thickness severity, or immunosuppressive treatments; therefore, we could not include this information in our analyses. Nonetheless, the comprehensive scope of this study remains a significant strength. Our stratified analyses by age, sex, and region provide a nuanced understanding of how mortality patterns evolved during the pandemic. These insights are critical for developing targeted public health strategies, reducing health disparities in high-risk populations, and understanding the broader indirect consequences of the pandemic on chronic disease management. More importantly, these findings can guide future healthcare planning and pandemic response strategies to protect high-risk groups and ensure that vulnerable patients continue to receive the care they need, even during crises. Building a healthcare system that can manage emerging infectious diseases while maintaining continuity of care for chronic conditions is critical for safeguarding public health.

## 5. Conclusions

Although the COVID-19 pandemic did not significantly increase overall excess mortality among patients with SSc, it had a substantial impact on specific subgroups. Elderly and female patients are particularly vulnerable, with heart failure and pneumonia emerging as the leading causes of death. Additionally, younger SSc patients experienced a significant rise in mortality compared to the pre-pandemic period, underlining the importance of targeted interventions for this high-risk group. To prevent similar outcomes in future public health crises, strengthening healthcare systems to ensure continuous care for patients with chronic illnesses is crucial. This can be achieved by decentralizing specialist services, expanding access to telemedicine, improving regional healthcare equity, and integrating pandemic preparedness into the management of chronic diseases. Protecting high-risk populations, such as individuals with systemic sclerosis (SSc), must become a priority in public health strategies, not only during emergencies but also in routine, equitable care delivery.

## Figures and Tables

**Table 1:** Comparison of all-cause mortality by period.

Factor	1 January 2015– 31 December 2019 (Pre-Pandemic)	1 January 2020– 31 December 2020 (Pandemic)	1 January 2021– 31 December 2021 (Pandemic)	1 January 2022– 31 December 2022 (Pandemic)
Whole population all-cause mortality				
Sex				
Total, *n* (%)	2,325,384 (100%)	492,226 (100%)	555,056 (100%)	586,839 (100%)
Male, *n* (%)	1,321,037 (56.81%)	279,650 (56.81%)	315,023 (56.76%)	330,366 (56.30%)
Female, *n* (%)	1,004,347 (43.19%)	212,576 (43.19%)	240,033 (43.24%)	256,473 (43.70%)
Age				
18–29 years, *n* (%)	73,790 (3.17)	13,383 (2.72)	13,425 (2.42)	13,386 (2.28)
30–39 years, *n* (%)	109,972 (4.73)	20,071 (4.08)	21,982 (3.96)	21,465 (3.66)
40–49 years, *n* (%)	215,416 (9.26)	42,904 (8.72)	47,641 (8.58)	46,485 (7.92)
50–59 years, *n* (%)	337,221 (14.50)	70,453 (14.31)	80,472 (14.50)	78,994 (13.46)
60–69 years, *n* (%)	426,170 (18.33)	93,348 (18.96)	107,212 (19.32)	109,652 (18.69)
≥70 years, *n* (%)	1,162,815 (50.01)	252,067 (51.21)	284,324 (51.22)	316,857 (53.99)

**Table 2:** Comparison of all-cause mortality, SSc-related death, death due to COVID-19, and the five most common causes of non-SSc-related death in SSc patients by period.

Factor	1 January 2015– 31 December 2019 (Pre-Pandemic)	1 January 2020– 31 December 2020 (Pandemic)	1 January 2021– 31 December 2021 (Pandemic)	1 January 2022– 31 December 2022 (Pandemic)
**All-cause mortality**				
Total, *n* (%)	3693 (100%)	1026 (100%)	976 (100%)	1105 (100%)
Sex				
Male, *n* (%)	1454 (39.37%)	437 (42.55%)	417 (42.72%)	468 (42.35%)
Female, *n* (%)	2239 (60.63%)	589 (57.35%)	559 (57.28%)	637 (57.65%)
Age				
18–29 years, *n* (%)	32 (0.86%)	10 (0.97%)	6 (0.61%)	7 (0.63%)
30–39 years, *n* (%)	110 (2.97%)	26 (2.53%)	10 (1.02%)	22 (1.99%)
40–49 years, *n* (%)	369 (9.99%)	105 (10.23%)	91 (9.32%)	78 (7.06%)
50–59 years, *n* (%)	977 (26.45%)	238 (23.19%)	232 (23.77%)	273 (24.71%)
60–69 years, *n* (%)	1212 (32.81%)	352 (34.30%)	337 (34.52%)	368 (33.30%)
≥70 years, *n* (%)	993 (26.88%)	295 (28.75%)	300 (30.73%)	357 (32.31%)
**SSc-related death**				
Total, *n* (%)	187	55	44	51
Sex				
Male, *n* (%)	67 (35.83%)	23 (41.82%)	25 (56.82%)	20 (39.22%)
Female, *n* (%)	120 (64.17%)	32 (58.18%)	19 (43.18%)	31 (60.78%)
Age				
18–29 years, *n* (%)	1 (0.53%)	0	0	1 (1.96%)
30–39 years, *n* (%)	12 (6.42%)	1 (1.82%)	1 (2.27%)	1 (1.96%)
40–49 years, *n* (%)	20 (10.70%)	8 (14.55%)	6 (13.64%)	4 (7.84%)
50–59 years, *n* (%)	70 (37.43%)	14 (25.45%)	11 (25.9%)	20 (39.22%)
60–69 years, *n* (%)	54 (28.88%)	25 (45.45%)	17 (38.64%)	18 (35.29%)
≥70 years, *n* (%)	30 (16.04%)	7 (12.73%)	9 (20.45%)	7 (13.73%)
**Death due to COVID-19**				
Total, *n* (%)	N/A	N/A	6	36
Sex	N/A	N/A		
Male, *n* (%)	N/A	N/A	2 (33.33%)	12 (33.33%)
Female, *n* (%)	N/A	N/A	4 (66.67%)	24 (66.67%)
Age	N/A	N/A		
18–29 years, *n* (%)	N/A	N/A	0	0
30–39 years, *n* (%)	N/A	N/A	0	6 (16.67%)
40–49 years, *n* (%)	N/A	N/A	0	0
50–59 years, *n* (%)	N/A	N/A	0	0
60–69 years, *n* (%)	N/A	N/A	2 (33.33%)	8 (22.22%)
≥70 years, *n* (%)	N/A	N/A	4 (66.67%)	22 (61.11%)
**Non-SSc related death (5 most common causes)**				
Heart Failure	368 (9.96%)	109 (10.62%)	104 (10.66%)	112 (10.14%)
Pneumonia	293 (7.93%)	88 (8.58%)	72 (7.38%)	108 (9.77%)
Malignant Neoplasms	197 (5.33%)	64 (6.24%)	74 (7.58%)	53 (4.80%)
Sepsis	197 (5.33%)	45 (4.39%)	46 (4.71%)	61 (5.52%)
Chronic kidney disease	113 (3.06%)	31 (3.02%)	24 (2.46%)	30 (2.71%)

SSc: systemic sclerosis, N/A: Data unavailable; relevant period predates the emergence of COVID-19.

**Table 3:** Analysis of Excess Mortality by time and age group.

Age Group	Expected Deaths	Observed Deaths	Excess Deaths	Excess 95%CI	*p*-Score
**Age 18–29 years**					
All-cause whole population mortality	42,550.72	40,194	−2356.72	−2761 to −1952	−5.54
SSC-related death					
1 January 2020–31 December 2020	3	10	7	4 to 10	233
1 January 2021–31 December 2021	4	6	2	−2 to 6	50
1 January 2022–31 December 2022	5	7	2	−2 to 6	41
Total COVID pandemic period	12	23	11	4 to 18	92
**Age 30–39 years**					
All-cause whole population mortality	58,899.01	63,518	4618.994	4143 to 5095	7.84
SSc-related death					
1 January 2020–31 December 2020	30	26	−4	−15 to 7	−13
1 January 2021–31 December 2021	34	10	−24	−35 to −13	−71
1 January 2022–31 December 2022	39	22	−17	−29 to −5	−44
Total COVID pandemic period	103	58	−45	−65 to −25	−44
**Age 40–49 years**					
All-cause whole population mortality	129,146.2	137,030	7883.847	7179 to 8588	6.1
SSc-related death					
1 January 2020–31 December 2020	102	105	3	−17 to 23	3
1 January 2021–31 December 2021	115	91	−24	−45 to −3	−21
1 January 2022–31 December 2022	129	78	−51	−73 to −29	−40
Total COVID pandemic period	346	274	−72	−108 to −36	−21
**Age 50–59 years**					
All-cause whole population mortality	222,081	229,919	7837.983	6914 to 8762	3.53
SSc-related death					
1 January 2020–31 December 2020	281	238	−43	−76 to −10	−15
1 January 2021–31 December 2021	318	232	−86	−121 to −51	−27
1 January 2022–31 December 2022	362	273	−89	−126 to −52	−25
Total COVID pandemic period	961	743	−218	−279 to −157	−23
**Age 60–69 years**					
All-cause whole population mortality	291,386.9	310,212	18,825.14	17,767 to 19,833	6.46
SSc-related death					
1 January 2020–31 December 2020	324	352	28	−7 to 63	9
1 January 2021–31 December 2021	359	337	−22	−59 to 15	−6
1 January 2022–31 December 2022	398	368	−30	−69 to 9	−8
Total COVID pandemic period	1081	1057	−24	−88 to 40	−2
**Age ≥ 70 years**					
All-cause whole population mortality	774,631	853,248	78,617.01	76,892 to 80,342	10.15
SSc-related death					
1 January 2020–31 December 2020	309	295	−14	−48 to 20	−4
1 January 2021–31 December 2021	362	300	−62	−99 to −25	−17
1 January 2022–31 December 2022	426	357	−69	−109 to −29	−16
Total COVID pandemic period	1098	952	−146	−211 to −81	−13

SSc: systemic sclerosis.

**Table 4:** Analysis of excess mortality by time and sex.

Population	Expected Deaths	Observed Deaths	Excess Deaths	Excess Deaths 95%CI	*p*-Score
**Female**					
All-cause whole population mortality	978,310	800,602	55,816	54,232 to 57,400	5.7
SSc-related death					
1 January 2020–31 December 2020	633	589	−44	−93 to 5	−7.0
1 January 2021–31 December 2021	713	559	−154	−206 to −102	−21.6
1 January 2022–31 December 2022	806	637	−169	−225 to −113	−21.0
Total COVID pandemic period	2152	1785	−368	−459 to −277	−17.1
**Male**					
All-cause whole population mortality	863,214.2	925,039	61,824.82	60,004 to 63,646	7.16
SSc-related death					
1 January 2020–31 December 2020	421	437	16	−24 to 56	3.8
1 January 2021–31 December 2021	478	417	−61	−104 to 56	−12.76
1 January 2022–31 December 2022	546	468	−78	−124 to −32	−14.3
Total COVID pandemic period	1445	1322	−123	−198 to −48	−8.5

SSc: systemic sclerosis.

## Data Availability

Data and materials are available from the corresponding author upon reasonable request.

## Abbreviations

ACR	American College of Rheumatology
COVID-19	Coronavirus disease 2019
dcSSc	Diffuse cutaneous systemic sclerosis
EULAR	European Alliance of Associations for Rheumatology
ICD-10	The International Classification of Diseases, Tenth Revision
lcSSc	Limited cutaneous systemic sclerosis
NHSO	The National Health Security Office
RA	Rheumatoid arthritis
SARS-CoV-2	Coronavirus 2
SD	Standard deviation
SLE	Systemic lupus erythematosus
SSc	Systemic sclerosis

## References

[1] Allanore Y., Simms R., Distler O., Trojanowska M., Pope J., Denton C.P., Varga J. (2015). Systemic sclerosis. Nat. Rev. Dis. Primers.

[2] Mehta B.K., Espinoza M.E., Hinchcliff M., Whitfield M.L. (2020). Molecular “omic” signatures in systemic sclerosis. Eur. J. Rheumatol..

[3] The COVID-19 Pandemic and Continuing Challenges to Global Health. https://www.who.int/about/funding/invest-in-who/investment-case-2.0/challenges.

[4] Khingchatturat N. (2022). Impact from COVID-19 to the changing of Thai health system. Public Health Policy Laws J..

[5] Fekadu G., Bekele F., Tolossa T., Fetensa G., Turi E., Getachew M., Abdisa E., Assefa L., Afeta M., Demisew W. (2021). Impact of COVID-19 pandemic on chronic diseases care follow-up and current perspectives in low resource settings: A narrative review. Int. J. Physiol. Pathophysiol. Pharmacol..

[6] Emmi G., Bettiol A., Mattioli I., Silvestri E., Di Scala G., Urban M.L., Vaglio A., Prisco D. (2020). SARS-CoV-2 infection among patients with systemic autoimmune diseases. Autoimmun. Rev..

[7] Gianfrancesco M., Hyrich K.L., Al-Adely S., Carmona L., I Danila M., Gossec L., Izadi Z., Jacobsohn L., Katz P., Lawson-Tovey S. (2020). Characteristics associated with hospitalisation for COVID-19 in people with rheumatic disease: Data from the COVID-19 Global Rheumatology Alliance physician-reported registry. Ann. Rheum. Dis..

[8] Chaisrimaneepan N., Thiravetyan B., Nakaphan P., Puchongmart C. (2025). The impact of systemic sclerosis on hospitalized COVID-19 patients: Analysis of the US nationwide inpatient sample (2021). J. Scleroderma. Relat. Disord..

[9] Bournia V.K., Fragoulis G.E., Mitrou P., Mathioudakis K., Tsolakidis A., Konstantonis G., Tseti I., Vourli G., Tektonidou M.G., Paraskevis D. (2023). Different COVID-19 outcomes among systemic rheumatic diseases: A nation-wide cohort study. Rheumatology.

[10] COVID-19 Cases|WHO COVID-19 Dashboard. Datadot. https://data.who.int/dashboards/covid19/cases.

[11] WTO|COVID-19 and Trade by Country. https://www.wto.org/english/tratop_e/covid19_e/covid_details_by_country_e.htm?country=THA.

[12] Mengjuan D., Handcock M.S., Blackburn B., Kee F., Biaukula V., Matsui T., Olowokure B. (2022). Tool for tracking all-cause mortality and estimating excess mortality to support the COVID-19 pandemic response. Western. Pac. Surveill. Response. J..

[13] Zarif A., Joy M., Sherlock J., Sheppard J.P., Byford R., Akinyemi O., Bankhead C.R., Deeks A., Ferreira F., Jones N. (2021). The impact of primary care supported shielding on the risk of mortality in people vulnerable to COVID-19: English sentinel network matched cohort study. J. Infect..

[14] Sloan M., Gordon C., Lever E., Harwood R., A Bosley M., Pilling M., Brimicombe J., Naughton F., Blane M., Walia C. (2021). COVID-19 and shielding: Experiences of UK patients with lupus and related diseases. Rheumatol. Adv. Pract..

[15] Ammitzbøll C., Andersen J.B., Vils S.R., Jørgensen C.M., Hauge E.M., Erikstrup C., Mikkelsen S., Thomsen M.K., Troldborg A. (2021). POS1171 The COVID-19 Pandemic Prompts Isolation and Behavioral Changes in Patients with Chronic Rheumatic Diseases Leading to Reduced Physical Activity, Increased Pain, Disease Activity, and Low Seroprevalence of SARS-CoV2 Antibodies. Ann. Rheum. Dis..

[16] Stronati G., Ribichini F., Benfaremo D., Dichiara C., Casella M., Dello Russo A., Guerra F., Moroncini G. (2021). Long term prognosis and cardiovascular complications of patients with systemic sclerosis-related cardiomiopathy. Eur. Heart J..

[17] Tyndall A.J., Bannert B., Vonk M., Airò P., Cozzi F., Carreira P.E., Bancel D.F., Allanore Y., Müller-Ladner U., Distler O. (2010). Causes and risk factors for death in systemic sclerosis: A study from the EULAR Scleroderma Trials and Research (EUSTAR) database. Ann. Rheum. Dis..

[18] Li J.X. (2022). Secular Trends in Systemic Sclerosis Mortality in the United States from 1981 to 2020. Int. J. Environ. Res. Public Health.

[19] Pacheco J., Crispi F., Alfaro T., Martínez M.S., Cuadrado C. (2021). Gender disparities in access to care for time-sensitive conditions during COVID-19 pandemic in Chile. BMC Public Health.

[20] Apipattarakul R., Foocharoen C., Netwijitpan S., Mahakkanukrauh A., Suwannaroj S., Limpawattana P., Nanagara R. (2018). Clinical characteristics and mortality rate of Thai elderly-onset systemic sclerosis. Clin. Exp. Rheumatol..

[21] Hughes M., Pauling J.D., Moore A., Jones J. (2021). Impact of COVID-19 on clinical care and lived experience of systemic sclerosis: An international survey from EURORDIS-Rare Diseases Europe. J. Scleroderma. Relat. Disord..

[22] Dorr N., Fennell P., Shapiro L. (2022). AB1398 Correlates of Cancelled Healthcare Appointments in Patients with Systemic Sclerosis During the COVID-19 Pandemic. Ann. Rheum. Dis..

[23] Foocharoen C., Ngamjarus C., Pattanittum P., Suwannaroj S., Pongkulkiat P., Onchan T., Wattanasukchai L., Chaiyarit J., Mahakkanukrauh A. (2023). Incidence and prevalence of systemic sclerosis in Thailand in year 2017-2020: A database from the Ministry of Public Health. Clin. Rheumatol..

[24] Li L., Serio J., Vosylis R., Sorgente A., Lep Ž., Zhang Y., Fonseca G., Crespo C., Relvas A.P., Zupančič M. (2023). Employment disruption and wellbeing among young adults: A cross-national study of perceived impact of the COVID-19 lockdown. J. Happiness. Stud..

[25] Hargreaves D.S., Greaves F., Levay C., Mitchell I., Kock U., Esch T., Denny S., Frich J.C., Struijs J., Sheikh A. (2015). Comparison of health care experienceanc access between young and older adults in 11 high-income countries. J. Adolesc. Health..

[26] Salazar de Pablo G., Vaquerizo-Serrano J., Catalan A., Arango C., Moreno C., Ferre F., Shin J.I., Sullivan S., Brondino N., Solmi M. (2020). Impact of coronavirus syndromes on physical and mental health of health care workers: Systematic review and meta-analysis. J. Affect. Disord..

[27] Baker M.A., Sands K.E., Huang S.S., Kleinman K., Septimus E.J., Varma N., Blanchard J., Poland R.E., Coady M.H., Yokoe D.S. (2022). The Impact of Coronavirus Disease 2019 (COVID-19) on Healthcare-Associated Infections. Clin. Infect. Dis..

[28] Zhao Y.H., Zhao L., Yang X.C., Wang P. (2021). Cardiovascular complications of SARS-CoV-2 infection (COVID-19): A systematic review and meta-analysis. Rev. Cardiovasc. Med..

